# Aboriginal Health Worker perceptions of oral health: a qualitative study in Perth, Western Australia

**DOI:** 10.1186/s12939-016-0299-7

**Published:** 2016-01-12

**Authors:** Angela Durey, Dan McAullay, Barry Gibson, Linda Slack-Smith

**Affiliations:** School of Dentistry, University of Western Australia, 35 Stirling Highway, Perth, 6009 WA Australia; School of Clinical Dentistry, University of Sheffield, 31 Claremont Crescent,, Sheffield, S10 2TA UK

**Keywords:** Australia, Aboriginal, Oral health, Inequity, Racism

## Abstract

**Background:**

Improving oral health for Aboriginal Australians has been slow. Despite dental disease being largely preventable, Aboriginal Australians have worse periodontal disease, more decayed teeth and untreated dental caries than other Australians. Reasons for this are complex and risk factors include broader social and historic determinants such as marginalisation and discrimination that impact on Aboriginal people making optimum choices about oral health. This paper presents findings from a qualitative study conducted in the Perth metropolitan area investigating Aboriginal Health Workers’ (AHWs) perceptions of barriers and enablers to oral health for Aboriginal people.

**Methods:**

Following extensive consultation with Aboriginal stakeholders, researchers conducted semi-structured interviews and focus groups across 13 sites to investigate AHWs’ perceptions of barriers and enablers to oral health based on professional and personal experience. Responses from 35 AHWs were analysed independently by two researchers to identify themes that they compared, discussed, revised and organised under key themes. These were summarised and interrogated for similarities and differences with evidence in the literature.

**Results:**

Key findings indicated that broader structural and social factors informed oral health choices. Perceptions of barriers included cost of services and healthy diets on limited budgets, attending services for pain not prevention, insufficient education about oral health and preventing disease, public dental services not meeting demand, and blame and discrimination from some health providers. Suggested improvements included oral health education, delivering flexible services respectful of Aboriginal people, oral health services for 0–4 year olds and role modelling of oral health across generations.

**Conclusion:**

Reviewing current models of oral health education and service delivery is needed to reduce oral health disparities between Aboriginal and non-Aboriginal Australians. Shifting the discourse from blaming Aboriginal people for their poor oral health to addressing structural factors impacting on optimum oral health choices is important. This includes Aboriginal and non-Aboriginal stakeholders working together to develop and implement policies and practices that are respectful, well-resourced and improve oral health outcomes.

## Background

Poor oral health in Aboriginal Australians is a significant concern, exacerbated by slow progress in reducing health disparities between Aboriginal and other Australians [1]. Evidence indicates that, despite dental caries being largely preventable, Aboriginal Australians have worse periodontal disease [2], more decayed teeth and untreated dental caries [3], with Aboriginal children having twice the rate of dental caries compared to non-Aboriginal children [4] and worse oral soft-tissue disease [5].

Various studies have identified risk factors contributing to poor oral health in this population including smoking, alcohol consumption [6] and diets high in sugar [7]. A recent study of urban Aboriginal people associated smoking and diabetes with severe periodontal disease [8]. Understanding how broader social determinants can undermine decisions about oral health is also important [9]. It avoids the trap of tacitly blaming Aboriginal people for their poor oral health because of ‘non-compliance’ with public health messages and instead recognises complex factors underpinning choice [10].

Evidence suggests lower oral health literacy is associated with worse periodontal disease [11], and limited knowledge of preventing dental disease, low socio-economic status and costly services can result in avoiding dental visits [12]. In Australia, oral health services are available in public, private and Aboriginal health service contexts. Barriers to good oral health and dental care identified by Aboriginal people include services disrespectful of cultural differences and/or with no Aboriginal Health Workers (AHWs) on staff [13]; cost, long waiting times, distance to services, especially for those living in remote areas [14], and limited access to healthy food [15]. An Aboriginal Health Worker is defined as a person of Aboriginal or Torres Strait Islander descent or who identifies as such and is accepted by the Aboriginal and Torres Strait Islander community; has a minimum qualification in Aboriginal and Torres Strait Islander primary health care and delivers care that is holistic and culturally safe [16].

Promoting oral health requires improving oral health literacy and involving Aboriginal people in designing strategies to prevent disease that are relevant and context specific [13]. A recent study of oral health care in Aboriginal Community Controlled Health Services (ACCHS) in New South Wales found that lifting the burden of oral disease in an Aboriginal community leaves more time to promote oral health [14]. However, even though dental care was sometimes accessible in ACCHS, the supply of oral health services fell very short of the demand and senior management identified that inadequate and inconsistent resources seriously undermined providing programs to treat disease and promote and sustain oral health care for Aboriginal people [14].

Relationships between health providers and their patients impact on health outcomes. A 1980s study in the US found that dentists preferred patients who were well educated, dentally sophisticated and calm, being more likely to be impatient with patients who were anxious [17]. More recent studies have shown that racism towards Aboriginal patients in health services, however inadvertent, has led to their reluctance to attend for treatment [18].

In Australia, whiteness is the ‘omnipresent norm’ against which differences from that norm, are measured and judged, often negatively (19: xix). White, English-speaking Australians have been privileged as a group since the colonisation and dispossession of Aboriginal Australians by the British in 1788. Indigenous rights and occupancy were ignored (“terra nullius”) and British authority determined policies and practices [19]. Being white provided structural advantage, usually invisible to those who were white, and reproduced inequities [19, 20] that continue to shape the lives of the privileged and the marginalised [21].

Interpersonal racism is being treated unfairly for being Aboriginal; internalised racism occurs when non-Aboriginal people’s racist assumptions and beliefs are incorporated into an Aboriginal person’s worldview; systemic or institutionalised racism is evident when differential power relations between Aboriginal and non-Aboriginal groups compromise Aboriginal people’s access to goods, services and opportunities [22]. Racism disproportionately affects Aboriginal Australians across sectors including education, employment [23] and health services where it often goes unchallenged and unreported [24] despite its harmful health effects [25, 26]. Government commitments to reduce discrimination against Aboriginal people often fall short and examples persist of Aboriginal health being under-resourced relative to need; inequitable treatment between Aboriginal and other patients; culturally inappropriate care; inadequate or no cross-cultural education of health care providers; market-driven health care provision; and models of individual care that prioritise diagnosis, treatment and cure rather than prevention [10]. One study called for physicians to critically reflect on their own assumptions about Aboriginal people and whether they were discriminatory based on race. The objective of such reflection was to avoid projecting any unconscious bias onto their patients that could negatively impact on their care [27].

The aim of this paper is to present findings from a qualitative study conducted in Perth, Western Australia, which investigated AHWs’ perceptions of barriers and enablers to oral health for Aboriginal people based on their professional and personal experience. The paper identifies whether AHWs’ responses support or challenge existing evidence and includes their suggestions for improving Aboriginal people’s oral health.

Ethics approval for this research was granted by the Human Research Ethics Committee at the University of Western Australia (RA/4/1/5792) and the Western Australian Aboriginal Health Ethics Committee (No.467).

## Methods

Following an extensive consultation process with key Aboriginal and non-Aboriginal stakeholders during 2013–2014, researchers followed up on contacts in various health services in Perth to invite AHWs to participate in semi-structured interviews or focus groups. A group discussion or interview was chosen depending on the workplace and the number of AHWs employed there. Where one AHW was employed an interview was offered, two or more AHWs were offered an interview together and a group discussion was offered where several AHWs worked at one site. No participant in a group discussion requested an individual interview. A follow up interview was conducted with one participant based on her past experience working with pregnant Aboriginal women. Thirty five health workers (28 females and seven males) from 13 different sites agreed to participate and were interviewed either by an Aboriginal or non-Aboriginal researcher or, where possible, by both. The lower number of male participants reflected the fact that there are nearly three times as many female AHWs employed as male in Australia [28]. Participants were given information about the project and invited to ask questions prior to signing consent forms to participate in interviews or focus groups. These were recorded, transcribed and imported into NVivo, a software programme to assist in the organisation and management of data during the analysis (http://www.qsrinternational.com).

Interview and focus group questions covered demographic characteristics (age, gender, highest education qualification, years working as an AHW, postcode) and topics including the meaning of oral health; issues facing Aboriginal people and their children related to oral health; the impact on oral health of diet, smoking and alcohol; challenges in promoting oral health and how oral health/dental services could be improved. Data saturation was reached after eight interviews and four focus groups, with no new relevant information emerging.

Responses were analysed independently by two researchers for themes related to barriers and enablers to oral health. Using an iterative approach, these were compared, discussed, revised and organised under key themes; any anomalies were noted in responses and data revisited and interpreted for meanings attributed to AHWs’ perceptions. Findings were then summarised and interrogated for similarities and differences and compared with existing evidence in the literature. Participant information was de-identified, abbreviated and classified numerically into Aboriginal Health Worker Female (AHWF) 1–28; Aboriginal Health Worker Male (AHWM) 1–7.

## Results

Interviews or focus groups involving male and female AHWs were held at participants’ workplaces. Responses highlighted how broader structural issues played a key role in choices around oral health and accessing services. We define structure as social, political and economic factors beyond the control of individuals yet which can adversely affect their health [29]. These included lack of education about oral health and disease prevention, public dental services not meeting the demand for care, discrimination by health providers and financial constraints that negatively impacted on decisions related to oral health. Suggested structural improvements included education on promoting oral health and disease prevention across the life span, free oral health services for 0–4 year olds and easier access to oral health services. Other enablers to oral health included health providers offering holistic, non-discriminatory, respectful care that was sensitive to the social and cultural contexts of Aboriginal people’s lives, encouraging role modelling of good oral health practice in families across generations and providing relevant and culturally sensitive materials to promote oral health.

### Structural barriers

Aboriginal people face numerous and well-documented structural barriers to maintaining oral health and accessing care including education, cost of services and discrimination by health providers. Most participants felt education about oral health and preventing disease was limited at best and often non-existent. This lack of knowledge had serious consequences for oral health including for those diagnosed with other comorbidities:

When we were diagnosed with diabetes, we weren’t even told that our teeth were an issue by the doctor (AHWF3).

This highlights the concept of inter-professional practice (e.g. medical/dental, dental/AHW) in improving overall health outcomes. Oral health was often not included in AHW training even though participants acknowledged that dental caries was a huge issue from pregnancy onwards in their communities. Dental disease was often compounded by structural issues beyond their control, not least the lack of oral health care in Western Australia for the 0–4 age group apart from private clinics and emergency services. Most participants acknowledged the benefits of free school dental services, accessible only to children in the year they turn five until Year 11 in high school. Cost for private and public services was a significant barrier to access:

Honestly it is too expensive. I have got three holes in my teeth, cavities, and I cannot afford to go to the dentist. Because my money is going on things that I think are more important like my house, power, everything else, food (AHWF8).

Accessing public dental services, even at ACCHS often required long waiting times and was compounded when transport was unavailable:

You have to be one of the first five in, in the morning otherwise you don’t get to see the dentist you have to wait for the next day. So they do struggle to get in there because you have to be in there by eight o’clock in the morning (AHWF14).

Cost and access to dental services were more burdensome for parents or carers looking after several children which often made attendance difficult, particularly without a creche:

More than likely you are not only going to be taking that child, but you will be taking other children with you. And when you are out for a couple of hours you have to have money for all of you because you have got the issue of food – it is a costly thing (AHWF13).

Even though participants understood the importance of preventing oral disease, accessing dental services for prevention was not a priority *‘because realistically how many people are going to go to the dentist if there is no pain?’ (AHWF7).* This point was further elaborated in the context of general health care:

If we look at access to health care – a lot of them won’t go to the doctor until something is seriously wrong. So if they are not going to do that in general day to day health, you’ve got Buckley’s of getting anyone going to see the dentist. ‘Oh, I need to go and see a dentist for an appointment just for my regular check-up’ – it’s not going to happen (AHWM3).

Other barriers to accessing care included perceived racism from health providers towards Aboriginal people and the apprehension this triggered:

You don’t want doctors and nurses judging you. They might not say it verbally but by looking at you (AHWF12).

Some participants felt patronised and judged by dental professionals that often led them to feel angry, humiliated, shamed and culturally unsafe:

Aboriginal people, when they walk into the dentist, it is that shame factor and they think they are being judged by the dentist, you know, ‘when was the last time you saw the dentist?’ ‘Do you brush your teeth? and ‘Your teeth aren’t healthy’. And these are adults – ‘And your gums aren’t healthy’ so the dentist is telling adults. And the adults are going home thinking ‘well, do I send my child there?’ (AHWF8).

For others, this kind of treatment exacerbated a trans-generational fear that their children would be removed because, as a parent, they felt health providers judged they had neglected to adequately care for their child’s teeth. One participant commented that a question asked of Aboriginal parents attending a hospital with their child was whether they were *‘under the Department of Child Protection’ (AHWF11).* She hoped this question was asked of all non-Aboriginal parents as well, but was unsure. The fear attributed to the question related to:

If they see it as neglect or something, they have got the power there to keep that kid away from you (AHWF12).

The legacy of colonisation and discrimination persists for Aboriginal people based on racial and cultural differences. Historically white, Anglo-Australian authorities forcibly removed Aboriginal children from their parents from the 1890s through to the 1970s [30], the so-called ‘stolen generation’. The negative effects of this are still experienced across generations in Aboriginal families [30] as reflected in the following comment that:

… you could go in there with your child and you could go out *without* your child. (AHWF11).

Participants’ responses indicated that health providers seemed to have little understanding of the historic and social context of their lives.

### Social factors

The importance of oral health emerged as a key theme that impacted on a sense of wellbeing:

People are concerned about their teeth – if they are painful, or they are ugly or they don’t want to smile because they have teeth missing (AHWF21).

Yet public health messages about the need for a healthy diet to prevent dental caries often lacked awareness of the lives of many Aboriginal families who juggled competing priorities including sharing food on a tight budget that stretched existing resources ‘*because you never know who is going to turn up at the door hungry’ (AHWF17):*

Fruit, veggies, it is all expensive. It is cheaper to buy a dollar packet of chips than it is to buy a bag of apples (AHWF22).

Many participants struggled to make ends meet, despite regular employment, and this impacted on their decision around oral health:

If we talk about Aboriginal people who work in the workforce, then you have to pay a lot more to go to the dentist. That would come last on your list of priorities not only because of all the other health issues you may have yourself, but within your family, you can’t afford to go to the dentist. That would be your last health thing that you would be concerned about. It would be better just to have your teeth ripped out (AHWM1).

Participants were concerned about alcohol and other drugs, particularly the use of methamphetamine or ‘speed’ in young people and the lack of information of its oral health effects:

…because you have a lot of young people where their teeth are absolutely rotting and falling away through speed (AHWM1).

Participants discussed Aboriginal families in their care who lived in unsettled, crowded environments, often moving between locations, making it difficult to buy, store and cook healthy food:

A lot of families that we see are not settled families, so maybe they are not living in their own accommodation, maybe they are sharing a house with a lot of other people. Just the stability – they might be home for one night or a week then they are over at Aunty’s house and staying for a week. So you have that on-the-go, moving around. It’s convenient just to grab the kids something to eat on the way to jump on the bus to go to Nan’s house instead of paying that money. You have got nowhere to store fruit and veggies or meat (AHWF13).

This type of environment also made it difficult to maintain oral health in other ways:

Something to do with lots of people sharing your house and not much private ownership of stuff. And even if you left your toothbrush in the bathroom, who do you know has used it? You can’t presume because it is yours and you leave it somewhere that no one is going to use it or play with it or it is going to end up outside. So that is pretty hard for people (AHWF10).

For many participants, toothache, when it did occur, often resulted in self-medication rather than visiting a dentist:

A lot of people I come across … they are happy to just continuously eat pain killers like they are going out of fashion …I’ve met a lot of people in the community who go ‘oh well, my gum is hurting and I am just going to take pain killers’ where they should be replacing them with antibiotics. (AHWF2)

Participants also commented they managed pain themselves to avoid going to the dentist:

Because if I rush straight off to the dentist, nine times out of ten I am going to get it pulled because that is the cheaper option … I don’t want my tooth pulled. I want to keep my teeth (AHWF22).

Nonetheless, dental extraction seemed common from a young age because of caries:

Seeing kids with a lot of decay and seeing kids having to have their teeth pulled at a young age. Having to go to surgery and have their baby teeth pulled out because they are rotten (AHWF13).

This method of treatment continued into adulthood:

For Aboriginal families we don’t go to the dentist until the tooth is ready to come out basically. You know you have got abscess, you put up with the pain, you put up with the, you know, you self-medicate yourself, only when your face is swollen …. and you see people with their face that is swollen because they have got an abscess … and that is children as well as adults.(AHWF12).

Participants also suggested various options to improve oral health in Aboriginal families.

### Improving oral health for Aboriginal people

Oral health promotion programs did exist and were effective up to a point, for example increasing its focus during a *‘dental health month’(AHWF7)* or AHWs implementing the ‘Lift the Lip’ [31] program for children under five years:

If they come in we can lift up their lip and check if there is plaque and we can see what their teeth are like … we can refer you and point you in the right direction but it’s the parent who has got to take ownership and do the rest of it (AHWF12).

All participants agreed about improving oral health education for their community from pregnancy onwards and presenting information in cost-effective ways that respected cultural differences. These included visual representations of oral health, photographs comparing healthy and decayed teeth and using flip charts. General health promotion needed to include oral health content starting with check-ups for pregnant women and continuing throughout the life-span for men and women.

Pictures, pamphlets with pictures. I always try to use pictures as much as I can and I yarn, that’s how I get through, because I’m out in the community (AHWF6)

I find a lot of those pictures that really show abnormal to normal – they sort of hit home. And too much writing in a pamphlet - you just need something on a small pamphlet that is to the point (AHWF2).

A one-size-fits-all approach to dissemination was considered ineffective and targeting information to those most in need was key:

It is the way it is publicised out in the community as well. A lot of people have smartphones that just hop on the internet but then there are a lot of people who aren’t familiar with the internet …You need to make sure it is filtering down to the different groups … but also include the people that are halfway there in terms of education. To me it appears that the people who need it the most are really not grasping where it is being advertised. Like it is not being advertised on their level. So, advertising filtering out to the people that are out there at that community level (AHWF2).

Employing Aboriginal Health Liaison Officers who focused specifically on oral health in health services was another suggestion:

Health liaison officers are all focused on diabetes and heart disease …where I think oral hygiene needs to have their own specific oral health liaison officers. If you had oral health liaison officers that actually went out into people’s homes and out in to the community and have done these assessments and education, imagine how much easier it would be; they are in their home already. They’re knocking on the door going into the home, how easy would it be to talk about oral hygiene? Because I know a lot of our mob don’t talk about oral hygiene in their homes (AHWF6).

Other suggestions included making oral health promotion fun and interactive by using educational games on smartphones and computers, reducing red tape to access services, more free dental services, incorporating oral health into Aboriginal child and adolescent general health check-ups:

So it is more of a preventative screening where you don’t have to be sick, you just go anyway and take children in. But I think there should be a similar thing around dental where every year, whether it is through Medicare or wherever, where people can go and get a screening test done every 12 months, and that is covered … Just like when people have their eyes checked you can go every 12 months – then it should be the same for dental (AHWF7).

Empowering parents to become role models of good oral health practice was another suggestion:

I think that’s the biggest thing. It is that role modelling. We need to get out into the community and get some role modelling and some good presentations (AHWF20).

Other ideas for promoting oral health included distributing dental packs (toothbrush, toothpaste and floss) at community events, festivals and open days and offering outreach services such as a dental van to visit kindergartens and sports events in the Aboriginal community:

So it goes to the areas or people that need it the most, having some sort of ‘meet us in the middle’ type thing …Outreach services have quite a lot of success. …We actually have a van that comes to our football club that is a travelling dentist so everyone lines up to have their mouthguard fitted. It’s great. As parents we think it is great. We don’t have to take a trip to the dentist because he is there (AHWF6).

Several participants thought oral health could be advertised more on TV and that schools could play a greater role in promoting oral health including posters in the classroom and regular teeth brushing:

I reckon school is probably the best I think – because if you educate the kids and get them into the routine of doing it. Sometimes there are issues around cost especially if you have five kids that you have to get toothbrushes and toothpaste and kids waste things as well … So knowing if they could get their teeth brushed at least one day a week from Monday to Friday in the full, that is better than not getting teeth brushed at all (AHWF7).

### Limitations

AHWs working in rural and remote locations were not interviewed. While this study was limited to Western Australia, we believe that the findings would, given the legacy of colonisation and discrimination, apply to other Aboriginal communities in Australia. We also suggest they could be applied to indigenous populations in other colonised countries such as Canada and New Zealand [32, 33].

## Discussion and conclusion

Participants’ responses indicate that oral health is important to Aboriginal people and current policy and practices are falling short in improving oral health outcomes. Key structural barriers identified by AHWs included insufficient education about oral health promotion and disease prevention. Private dental practice was considered out of reach financially for many Aboriginal people and public dental services were not meeting their oral health needs, were often hard to access without transport, incurred a cost and had no facilities for parents or carers with babies and young children. For some, the attitudes of health providers towards Aboriginal patients were also perceived as discriminatory. Dentists were seldom visited for check-ups and prevention. Instead, dental care was generally accessed only in an emergency when there was severe pain. Where possible, participants managed their own dental pain with analgesia and avoided dental visits partly from fear their teeth would be extracted. Given the prevalence of dental caries in many Aboriginal people, this was a reality and dental visits often resulted in tooth extraction, a cheaper option than tooth restoration according to some participants.

Other barriers included competing socio-economic priorities that impacted on decisions related to oral health for Aboriginal people. This suggests that improving oral health outcomes rests on more than ‘compliance’ with public health messages and implementing evidence-based interventions [10]. Instead, promoting and maintaining oral health is contingent on addressing a range of complex structural and social factors that are often ignored yet play a key role in decisions about oral health in this population. Our findings suggest that changing practice to promote oral health and prevent disease in Aboriginal people must be a shared responsibility between Aboriginal and non-Aboriginal Australians where non-Aboriginal policy makers and health care providers seek to understand the lived experience of Aboriginal Australians rather than making negative assumptions, judgements or inferring blame [34]. Locating poor oral health in Aboriginal people within a broader systemic framework reveals that current health care can undermine rather than promote oral health in this population and may not be respectful of cultural differences [27]. However, health providers and policy makers may be reluctant to examine their role in disadvantaging Aboriginal clients, not least if it suggests they are part of the problem [35].

AHW participants also suggested structural improvements such as disease prevention across the life span from pregnancy and education on how to promote oral health. This would include developing materials for oral health promotion that are relevant and culturally sensitive. Other suggestions included free oral health services for 0–4 year olds, inter-professional practice where oral health was part of general health checks, encouraging trans-generational role modelling of good oral health care, facilitating access to oral health services and ensuring services were respectful of Aboriginal people. This solution focused approach might also include health providers critically examining their own assumptions about Aboriginal people that could detrimentally impact on their care [10, 36] and adopting a holistic, non-discriminatory approach to care that is sensitive to Aboriginal peoples’ social and cultural contexts.

Participants thought a one-size-fits-all approach to dental services for Aboriginal people was inappropriate and required a more flexible, inter-professional model of education, prevention and treatment. This includes reviewing the current model of dental education for its ability to deliver care that is context specific, without prejudice and respectful of racial and cultural differences. Adopting a solution focused approach is a step towards providing a foundation for Aboriginal and non-Aboriginal stakeholders to work together in partnership to develop and implement policies and practices that are relevant to Aboriginal people, well-resourced and translate into sustained improvements to their oral health outcomes.

## References

[1] AIHW (2014). Mortality and life expectancy of Indigenous Australians: 2008 to 2012. Cat. no. IHW 140.

[2] Kapellas K, Skilton M, Maple-Brown L, Do L, Bartold P, O’Dea K (2014). Periodontal disease and dental caires among Indigenous Australians living in the Northern Territory, Australia. Aust Dent J.

[3] Jamieson L, Sayers S, Roberts-Thomson K (2010). Clinical oral health outcomes in young Australian Aboriginal adults compared with national-level counterparts. Med J Aust.

[4] Christian B, Blinkhorn A (2014). A review of dental caries in Australian Aboriginal children: the health inequalities pperspective. Rural Remote Health.

[5] Slack-Smith L, Read A, Colvin L, Leonard H, Kilpatrick N, McAullay D (2011). Total population investigation of dental hospitalizations in Indigenous children under five years in Western Australia using linked data. Aust Dent J.

[6] AIHW (2006). Australia’s health 2006.

[7] Jamieson L, Roberts-Thomson K, Sayers S (2010). Dental caries risk indicators among Australian Aboriginal young adults. Community Dent Oral Epidemiol.

[8] Roberts-Thomson K, Do L, Bartold P, Daniels J, Grosse A, Meihubers S (2014). Prevalence, extent and severity of severe periodontal destruction in an urban Aboriginal and Torres Strait Islander population. Aust Dent J.

[9] Kleinberger J, Strikhouser S (2014). Missing teeth: Reviewing the sociology of oral health and healthcare. Sociol Compass.

[10] Durey A, Thompson SC (2012). Reducing the health disparities of Indigenous Australians: Time to change focus. BMC Health Serv Res.

[11] Wehmeyer M, Corwin C, Guthmiller J, Lee J (2014). The impact of oral health literacy on periodontal health status. J Public Health Dent.

[12] Jones K, Parker E, Jamieson L (2014). Access, literacy and behavioural correlates of poor self-rated oral health amongst an Indigenous South Australian population. Community Dent Health.

[13] Jamieson L, Parker E, Richards L (2007). Using qualitative methodology to inform an Indigenous-owned oral health promotion initiative in Australia. Health Promot Int.

[14] Campbell M, Hunt J, Walker D, Williams R (2015). The oral health care experiences of NSW Aboriginal Community Controlled Health Services. Aust N Z J Public Health.

[15] Williams S, Jamieson L, MacRae A, Gray C (2011). Review of Indigenous oral health. Aust Indig Health Bull.

[16] HWA (2011). Growing our future: The Aboriginal and Torres Strait Islander health worker project final report.

[17] Corah N, O’Shea R, Bissell GD (1986). The dentist-patient relationship: mutual perceptions and behaviours. J Am Dent Assoc.

[18] Shahid S, Finn L, Thompson SC (2009). Barriers to participation of Aboriginal people in cancer care: Communication in the hospital setting. Med J Aust.

[19] Moreton RA (2009). Talkin’ up to the white woman.

[20] Pease B (2010). Undoing privilege: Unearned advantage in a divided world.

[21] Frankenberg R (1993). The social construction of whiteness Minneapolis: University of Minnesota Press.

[22] Paradies Y, Cunningham J (2009). Experiences of racism among urban Indigenous Australians: findings from the DRUID study. Ethnic Racial Stud.

[23] Walter M (2008). Lives of diversity: Indigneous Australia. Occasional Paper 4/2008. Census Series #2.

[24] Johnstone M-J, Kanitsaki O (2009). The spectrum of ‘new racism’ and discrimination in hospital contexts. Collegian.

[25] Larson A, Coffin J, Gilles M, Howard P (2007). It’s enough to make you sick: the impact of racism on the health of Aboriginal Australians. Aust N Z J Public Health.

[26] Ziersch A, Gallaher G, Baum F, Bentley M (2011). Responding to racism: Insights on how racism can damage health from an urban study of Australian Aboriginal people. Soc Sci Med.

[27] Durey A, Thompson SC, Wood M (2011). Time to bring down the twin towers in poor Aboriginal hospital care: Addressing institutionalised racism and misunderstandings in communication. Intern Med J.

[28] Health Workforce Australia (2014). Australia’s Health Workforce Series – Aboriginal andTorres Strait Islander Health Workers / Practitioners in focus.

[29] White K (2002). An introduction to the sociology of health and illness.

[30] National Inquiry into the Separation of Aboriginal and Torres Strait Islander Children from their Families (1997). Bringing them home.

[31] SA Dental Service. Life the lip: six months to five years. Adelaide: Government of South Australia, SA Health; 2008.

[32] Aboriginal Healing Foundation (2002). The Healing Has Begun: An operational update from the Aboriginal Healing Foundation.

[33] Ramsden I (2002). Cultural safety and nursing education in Aotearoa and Te Waipounamu [PhD]: Victoria University.

[34] Durey A, Wynaden DG, Thompson SC, Davidson P, Bessarab D, Katzenellenbogen JM (2012). ‘Owning solutions’: A collaborative model to improve quality in hospital care for Aboriginal Australians. Nurs Inq.

[35] Hutnik N, Gregory J (2008). Cultural sensitivity training: Description and evaluation of a workshop. Nurse Educ Today.

[36] Pitner R, Sakamoto I (2005). The role of critical consciousness in multicultural practice: Examining how its strength becomes its limitation. Am J Orthopsychiatry.

